# A Novel Technology for Targeted Brain Temperature Management

**DOI:** 10.1007/s12028-023-01800-7

**Published:** 2023-07-27

**Authors:** Andrea Lavinio, Erta Beqiri, Ketan Kataria

**Affiliations:** 1 Cambridge University Hospitals NHS Foundation Trust Addenbrooke’s Hospital, BOX 1 Hills Road, Cambridge, CB2 0QQ UK; 2 Brain Physics Laboratory Division of Neurosurgery, Department of Clinical Neurosciences, University of Cambridge, Cambridge, UK

**Keywords:** Target temperature management, Selective brain cooling, Traumatic brain injury, Intracranial pressure, Neuroprotection

Many clinical studies have failed to translate preclinical neuroprotective benefits of therapeutic hypothermia into improved outcomes for patients with acute brain injury. Clinical evidence points toward potential harm when therapeutic hypothermia is applied in the context of mild to moderate intracranial hypertension following traumatic brain injury [1] or when hypothermia is induced with intravascular cooling devices following stroke [2, 3].

A possible explanation for this could be sought in two inherent limitations of the technologies that are most commonly used in our neurology intensive care units (ICUs) (i.e., cooling blankets and intravascular devices). These limitations are the lack of brain selectivity and limited portability. The first shortcoming of the commonly available targeted temperature management (TTM) technologies is that they induce whole-body hypothermia rather than selectively targeting the brain. It is possible that the systemic complications associated with whole-body hypothermia, such as low respiratory tract infections, may outweigh its neuroprotective benefits. The other potential flaw of these technologies is that they tend to be bulky and have limited portability. It is well described that rapid temperature changes and fast rewarming are detrimental to the injured brain [4]. Because patients with brain injury are transported for emergency computerized tomography imaging or surgery when the brain is in its most vulnerable state, it is reasonable to assume that disconnection from cooling devices for transport and accidental rapid rewarming may cause harm.

Our group has developed a technology [5] that allows selective brain temperature management with a highly portable device, thus maximizing neuroprotection while minimizing systemic side effects. Thanks to its portability, the technology may also allow the early application of TTM in out-of-hospital settings (i.e., ambulances) and continuity of care when patients are transferred out of the ICU for imaging or emergency surgery.

The primary objective of this study was to evaluate the safety of delivering selective brain temperature management in humans using a cervical external cooling device. The primary end point focused on maintaining intracranial pressure below 20 mm Hg, serving as a crucial measure of safety. Additionally, a secondary objective was to assess the brain-to-core temperature differential as an indicator of the feasibility of implementing a selective approach to brain temperature management.

These specific end points were carefully chosen to provide preliminary evidence essential for designing a larger in-human clinical trial. The primary aim was to demonstrate that the application of the cervical external cooling device does not pose the risk of venous obstruction or precipitate intracranial hypertension, particularly in patients with depleted intracranial volume buffering reserve. Furthermore, we aimed to investigate whether the device offers a higher level of brain selectivity compared with traditional devices. By addressing these objectives, our study aimed to lay the foundation for further studies and advancements in selective brain temperature management (Fig. 1).

**Fig. 1:**
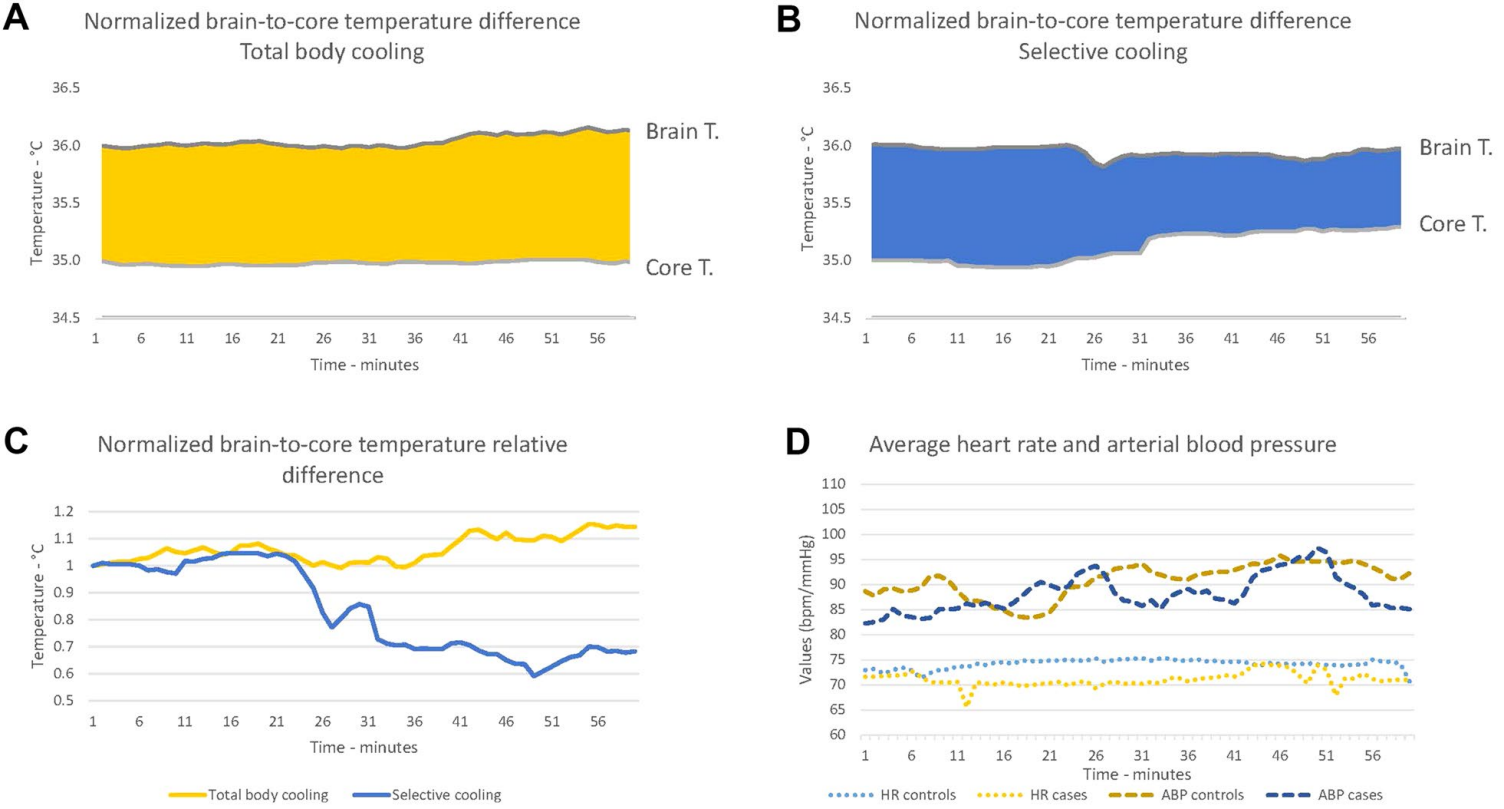
Normalized brain temperature (Brain T.) and core temperature (Core T.) (°C) over 60 min of treatment during systemic cooling (**a**) and selective cooling (**b**). Difference in the area under the curve (AUC) of the normalized brain-to-core temperature relative difference over 60 consecutive minutes of treatment in controls and cases (**c**). Heart rate (HR) and mean arterial blood pressure (ABP) for both groups over the same period (**d**)

Here, we report the first-in-human study of the technology, sponsored by a grant from the National Institute for Health and Care Research Brain Injury MedTech Co-operative.

An external temperature management system was designed for this study. The system can deliver 240 W of cooling power through two active elements connected to the surface of the neck by a polymeric collar with pouches filled with distilled water, providing a heat exchange interface and improving patient comfort (Fig. 2). The physician sets the target skin temperature according to a preset surface temperature to ensure an optimal temperature gradient for heat extraction.

**Fig. 2:**
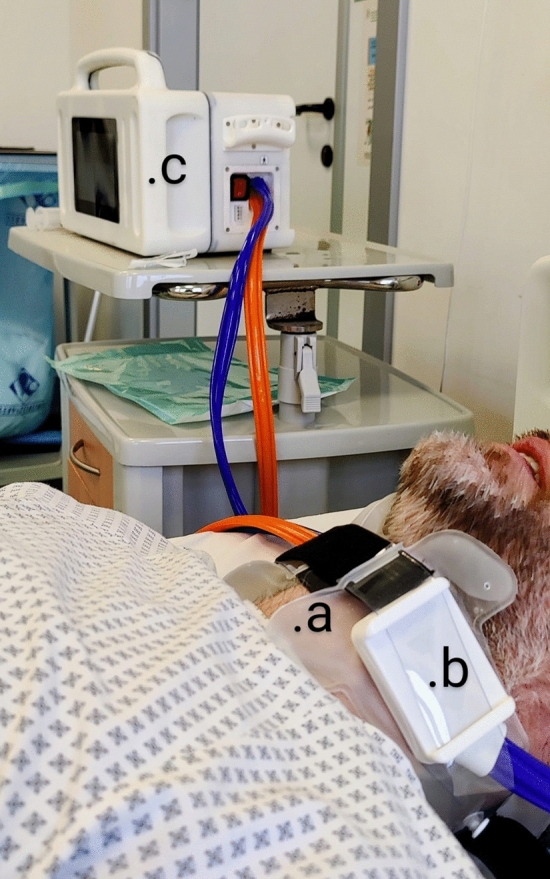
Picture depicting the prototype in standard configuration. Image taken during usability testing on a healthy volunteer for regulatory clearance (CE marking). **a,** A patient-device interface consisting of a fluid filled, single-use, biocompatible collar designed to maximize heat exchange while minimizing the risk of venous outflow impairment; **b,** an active element, consisting of a thermoelectric module, controlled by a dedicated control unit (**c**) (Specifications: dimensions: L: 35 cm; H: 27 cm; W: 20 cm; Weight: 7.5 kg; Maximum cooling power: 240 W; Maximum absorption: 400 W)

After receiving approval from the National Health Service, Health Research Authority, Research and Development Office (Integrated Research Application System ID: 248,587) and safety clearance from the local clinical engineering office, we conducted an open label, randomized, case–control study of ten comatose patients at the neurocritical care unit of the Cambridge University Hospital (Cambridge, UK) from July 2019 to November 2022.

We included adult patients with a clinical indication for invasive intracranial pressure (ICP) monitoring and brain temperature monitoring, as well as intracranial hypertension refractory to tier 1 and tier 2 treatments, indicating the need for TTM to a temperature below 36.5 °C as per local protocols. Patients were randomly assigned to receive either standard care (controls) or standard care plus the use of the prototype (cases) using preprepared concealed envelopes. Informed consent was sought from a consultee (i.e., next of kin).

For the control group, tier 1 and tier 2 ICP-lowering strategies (i.e., sedation, mechanical ventilation, inotropic support, osmotherapy, controlled normothermia) and TTM using an external cooling blanket (Arctic Sun 5000) were used according to local clinical protocols. Temperature targets were set by the treating physician, with the main outcome being an ICP below 20 mm Hg.

For the case group, tier 1 and tier 2 ICP-lowering strategies and temperature management delivered using the purpose-built collar prototype as a first-line method were used. The use of the external cooling blanket was allowed as clinically indicated prior to consent and randomization. A whole-body cooling blanket was also used as a rescue treatment if the treating physician felt that the control of the patient’s temperature or ICP using the Seletherm prototype was not adequate or in any cases of prototype malfunctions or safety concerns.

ICP was continuously monitored using intraparenchymal probes (Codman Microsensor). Brain temperature was continuously monitored using a combined intraparenchymal brain oximeter and brain temperature monitor (Licox, Integra). Brain monitoring probes were positioned in the right frontal lobe using a triple-access device. Core temperature was monitored using esophageal temperature probes. Data were digitized and captured continuously using ICM+ software (ICM+ ; Cambridge Enterprise Ltd, Cambridge, UK) at a sampling rate of 1 min (brain oximetry) or 240 Hz (all the other variables). All data are stored for further analysis and validation within the ICM+ platform, Academic Neurosurgery, University of Cambridge (https://icmplus.neurosurg.cam.ac.uk/). ICM+ was used to process the physiological measurements as follows: artifacts were automatically and manually removed; minute-by-minute values were calculated for each variable; and the pressure reactivity index was used (as previously described [4]) as a surrogate measure for cerebrovascular reactivity.

A period of 60 consecutive minutes corresponding to the treatment period was selected for all patients in both groups. Data collection and analysis was supervised by EB, a clinical researcher with no ties with Neuronguard S.R.L.

Nominal variables are presented as percentages. Continuous variables are presented as medians with interquartile ranges. We normalized brain and core temperatures by the respective initial temperatures and calculated the area under the curve (AUC) of the brain-core temperature gradient. We used the Wilcoxon rank-sum test to assess differences between two continuous variables.

ICP control was quantified as the total number of 1 min epochs with an ICP > 20 mm Hg and calculated as the AUC for ICPs > 20 mm Hg (mm Hg × min), normalized for the total duration of monitoring for comparison (mm Hg). Brain-to-body temperature differentials are expressed in °C.

Furthermore, we compared average brain oximetry and pressure reactivity index values between the two groups as a safety assessment.

The clinical experimental setting was designed to minimize the impact of confounding external factors that could potentially distort the signal-to-noise ratio by affecting ICP, brain temperature, and core temperature independently from the cooling devices used. Special care was taken to mitigate the influence of nursing maneuvers, pharmacological or physical interventions, and changes in environmental conditions such as temperature, humidity, or sunlight. Strict clinical supervision by experienced physicians (AL, KK) and independent technical support ensured safety and adherence to protocol. All patients were sedated, paralyzed and mechanically ventilated. To ensure optimal data collection and interpretation, data were specifically collected during steady-state conditions, deliberately selecting a period free from interventions that could impede the accurate measurement and analysis of data. These comprehensive measures, which limited the observation period to 60 min, were implemented to minimize external interference and optimize the reliability of the study results. By rigorously controlling the experimental conditions, we aimed to provide robust and credible evidence regarding the safety and feasibility of selective brain temperature management using the cervical external cooling device.

Ten patients with traumatic brain injury (seven men and three women) admitted to the Neurosciences and Trauma Critical Care Unit at Addenbrooke’s Hospital in Cambridge, UK, were enrolled in this study. All patients had a diagnosis of severe traumatic brain injury and intracranial hypertension refractory to tier 1 and tier 2 treatments, indicating TTM to a temperature < 36.5 °C. Patient demographics, injuries, and Glasgow Coma Scale scores at admission to and discharge from the ICU are summarized in Table 1.

**Table 1:** Patient demographics are reported for each group

Parameter	Control group	Case group
Sex	3 Male patients, 2 female patients	4 Male patients, 1 female patient
Age (y)	55 (27–55)	36 (26–38)
BMI (kg/m^2^)	24.1 (21.6–26.8)	26.6 (26.0–27.5)
GCS at the scene	4 (3–6)	3 (3–8)
GCS at admission	3	3
Diagnosis at admission (%)		
tSAH	30.8	33.3
DAI		22.3
SDH	30.8	22.2
EDH		11.1
ICH	7.6	
MLS	15.4	
Contusion	15.4	11.1
Outcome at discharge (%)	Alive, 100	Alive, 80
GCS at discharge	11 (9–11)	12 (9–14)
ICU LOS (d)	25 (24–26)	27 (21–29)

*BMI* Body mass index, Contusion, cerebellar or bifrontal contusion, *DAI* Diffuse axonal injury, *EDH* Epidural hematoma, *GCS* Glasgow coma scale, *ICH* Intracerebral hemorrhage, *ICU* Intensive care unit, *LOS* Length of stay, *MLS* Midline shift, *SHD* Subdural hematoma, *tSAH* Traumatic subarachnoid hemorrhage

ICP was well controlled in all patients, with average ICP values below 16 mm Hg. In all patients, the treating physician was satisfied with the control of temperature and ICP delivered by the prototype and cooling blanket, as no rescue treatment was necessary in either group.

The median (interquartile range) brain temperature at the end of the observation period was 36.5 °C (from 36.4 to 36.9 °C) in the control group and 36.2 °C (from 35.1 to 37.0 °C) in the case group. The median core temperature was 35.2 °C (from 34.9 to 36.2 °C) in the control group and 36.0 °C (from 34.3 to 36.4 °C) in the case group. The brain and core temperature, ICP, brain oximetry, and pressure reactivity index values are summarized in Table 2.

**Table 2:** Intracranial pressure and brain oximetry were controlled within the therapeutic targets throughout the treatment period in both cases and controls

Parameter	Control group	Case group	*p* Value
Intracranial pressure (mm Hg)	13.3 (IQR 10.7 to 16.6)	13.1 (IQR 9.8 to 13.5)	0.7540
Brain oximetry (mm Hg)	20.7 (IQR 17.5 to 29.7)	21.2 (IQR 20.9 to 30.8)	0.2967
PRx	− 0.01 (IQR − 0.05 to 0.24)	− 0.17 (IQR − 0.25 to − 0.11)	0.0283

The pressure reactivity index values indicated preserved cerebrovascular reactivity (< 0.2) in both case and control groups, with a statistically significant improvement in cerebrovascular reactivity parameters associated with selective brain cooling

IQR, interquartile range, PRx, xxx

The normalized brain-core temperature gradient AUC showed a statistically significant reduction of 29.85% in the case group compared with the control group: the median AUC was 48.06 (from 45.04 to 61.44) in the case group and 68.50 (from 68.48 to 68.85) in the control group (*p* value 0.0283; Fig. 1).

ICP, brain oximetry, and pressure reactivity index values were stable in both groups over the cooling period and within the safety ranges; the case group showed a slightly more favorable brain pressure reactivity index value (*p *= 0.0283; Table 2).

This first-in-human pilot study demonstrates the feasibility of delivering selective brain temperature management using brain and core temperature monitoring while applying active cooling pads to the neck, replicating the findings of earlier animal studies [5].

A reduced brain-to-core temperature gradient in patients treated with the collar prototype indicates selective brain cooling in the controlled setting of this study. Further clinical studies will be needed to confirm whether selective brain temperature management translates into improved neuroprotection and a reduction in the incidence and severity of systemic side effects associated with such treatment. The main limitation of this pilot study is the relatively small number of included patients and the short duration of observation, which may limit the generalizability of these preliminary findings. The group intends to use these findings as the basis for a larger clinical trial.

A recent review by Kendall et al. [6] evaluated 16 studies involving a total of 480 patients to assess temperature gradients between brain temperature and core temperature in various clinical settings. The review highlighted a high level of clinical and statistical heterogeneity among the included studies. Factors such as measurement device, physiological changes, local protocols, and medical or surgical interventions contributed to the variability in temperature gradients. The wide range of temperature gradients reported, spanning from − 1.29 to + 1.1 °C, underscores the diverse nature of these measurements in different patient populations and clinical scenarios. In our study, conducted within highly standardized settings, we observed a reduction in the brain-to-core temperature gradient with selective brain temperature management. Although our findings contribute to the existing literature, it is important to recognize its controlled settings and the limitations of its generalizability.

Cerebrovascular reactivity was preserved within the normal range in patients treated with systemic and selective neck cooling. There was a small but statistically significant trend toward improved cerebrovascular reactivity in patients treated with the cooling collar. This observation, in conjunction with satisfactory ICP control in both groups, provides reassurance that the cooling collar does not adversely affect venous outflow or cerebral blood flow.

Controlled normothermia and the prevention of neurogenic fever using automated devices are cornerstones of modern neuroprotection following hemorrhagic and ischemic stroke. TTM is the standard of care for anoxic-ischemic encephalopathy following cardiac arrest. The range of potential applications of a highly portable device capable of delivering selective brain cooling—theoretically maximizing the benefits of hypothermic neuroprotection while minimizing its side effects—may include traumatic brain injury and stroke.

### Supplementary Information

Below is the link to the electronic supplementary material.

Supplementary file1 (PDF 1073 KB)

## Clinical Trial Registration

National Health Service Health Research Authority, SELETHERM 1.2 IRAS ID 248587, https://www.hra.nhs.uk/planning-and-improving-research/application-summaries/research-summaries/seletherm-12/.
