# Supplementary material for: A Novel Technology for Targeted Brain Temperature Management
Source: Neurocrit Care. 2023 Jul 27;40(2):785–90. doi: 10.1007/s12028-023-01800-7 (PMC10959802; doi:10.1007/s12028-023-01800-7)
Supplement: Supplementary file 1 — Supplementary file1 (PDF 1073 KB) [file 12028_2023_1800_MOESM1_ESM.pdf]

Full Study Title: Selective brain temperature management after traumatic brain injury - SELETHERM

IRAS Ref: 248587

Date and Version No: 4th of January 2019, version 1.2

Chief Investigator:	Dr Andrea Lavinio - a.lavinio@addebrookes.nhs.uk – Addenbrooke's Ext. 216404<br>07960614932
Investigators:	Dr Andrea Lavinio / Dr Enrico Giuliani / Dr Luke Terrett
Sponsor:	Cambridge University Hospitals NHS FT

AMENDMENT HISTORY

**Amendment No.**	**Protocol Version No.**	**Date issued**	**Author(s) of changes**	**Details of Changes made**
1	1	5/9/2018	A LAVINIO	First draft
2	1.1	28/9/2018	A LAVINIO	Sample size, inclusion criteria, data collection and analysis
3	1.2	4/1/2019	A LAVINIO	Specified data retention period

List details of all protocol amendments here whenever a new version of the protocol is produced.

SIGNATURE PAGE

The undersigned confirm that the following protocol has been agreed and accepted and that the Chief Investigator agrees to conduct the study in compliance with the approved protocol and will adhere to the principles outlined in the Declaration of Helsinki, the Sponsor's SOPs, and other regulatory requirement.

I agree to ensure that the confidential information contained in this document will not be used for any other purpose other than the evaluation or conduct of the investigation without the prior written consent of the Sponsor

I also confirm that I will make the findings of the study publically available through publication or other dissemination tools without any unnecessary delay and that an honest accurate and transparent account of the study will be given; and that any discrepancies from the study as planned in this protocol will be explained.

For and on behalf of the Study Sponsor:

Signature: .... Date: ....../....../......

Name (please print):

Andrea Lavinio

Position: Clinical Lead, NCCU, Addenbrooke's

....

Chief Investigator:

Signature: .... Date: 04/01/2019

Name: (please print):

Andrea Lavinio

KEY STUDY CONTACTS

**Chief Investigator**	Dr Andrea Lavinio<br>Cambridge University Hospital<br>Hills Road - Box 1<br>CB2 0QQ<br>a.lavinio@addebrookes.nhs.uk
**Study Co-ordinator**	Dr Andrea Lavinio<br>Cambridge University Hospital<br>Hills Road - Box 1<br>CB2 0QQ<br>a.lavinio@addebrookes.nhs.uk
**Sponsor**	Cambridge University Hospitals NHS FT
**Joint-sponsor(s)/co-sponsor(s)**	None
**Funder(s)**	NIHR Brain Injury MedTech Co-operative<br>Dept. of Clinical Neuroscience<br>University of Cambridge<br>Box 167 - Cambridge Biomedical Campus<br>CB2 0QQ<br>01223336944<br>info@brainmic.org<br><br>Neuron Guard S.r.l.<br>via L. Castelvetro 19<br>41124 Modena (MO)<br>Italy<br>info@neuronguard.com

SYNOPSIS

**Study Title**	Selective brain temperature management after traumatic brain injury – SELETHERM
**Internal ref. no.**	ALEG - 01
**Study Design**	Interventional, randomised, first-in-human, proof-of-concept pilot study comparing two methods of brain temperature management following brain injury: total body cooling (standard care) and selective cervical cooling (experimental).<br><br>Target temperature management delivered by means of systemic cooling is standard of care for brain protection following severe TBI. This is typically delivered by means of body surface cooling and infusion of ice-cold crystalloids. Systemic cooling is known to be associated with cardiac, respiratory, renal and infective complications.<br><br>This study will test the feasibility and safety of cervical cooling to deliver selective brain cooling in a small number of brain injured patients – as instigated by the NIHR i4i panel.<br><br>Brain temperature and systemic temperature will be monitored as part of standard clinical care. Temperature management will be delivered by means of standard care (systemic cooling) or by selective cervical cooling using a purpose-built prototype, developed and provided by Neuronguard.<br><br>The primary outcomes will be safety of the experimental strategy, its ability to maintain brain temperature within temperature targets set by the treating physician and the proof-of-concept that cervical cooling can induce selective brain cooling (i.e. quantified as the brain-body temperature gradient).<br><br>If cervical cooling shows the ability to deliver selective brain temperature, the strategy will be tested in subsequent clinical study on a larger sample to describe its safety profile against systemic cooling.
**Study Participants**	Adult, comatose patients admitted to NCCU with acute brain injury as a result of brain trauma with clinical indication for invasive monitoring of brain physiology (i.e. intracranial pressure, cerebral oximetry and parenchymal brain temperature, cerebral microdialysis) as part of their standard clinical management and indication for target temperature management at temperature target < 35 C. Patients with devastating injuries not expected to survive 24 hours will be excluded from the study.
**Planned Sample Size (if applicable)**	At least 10 patients will be randomly allocated to two groups using pre-allocated sealed identical envelopes.<br>**Standard care (5 patients):** standard clinical protocol;

	**Experimental care (5 patients):** standard clinical care with cervical cooling and systemic cooling allowed as a rescue procedure if temperature targets not achieved;
**Planned Study Period**	90 days – the trial will be interrupted early if there is any concern regarding the safety of the device.
**Primary Objective**	**Safety.** Describe number and severity of complications associated with induction and of hypothermia using selective cervical cooling:<br>• failure to maintain the brain within target temperature;<br>• number of instances where external cooling blankets or ice-cold saline had to be used as a rescue strategy to achieve target temperature;<br>• duration and severity of cerebral hyperthermia;<br>• any other adverse event;
**Secondary Objectives**	**Proof of concept.** Describe the ability of the device to deliver selective brain cooling, and its effectiveness:<br>• differential brain-to-core temperature as evidence of selective cooling (between-group comparison);<br>• brain temperature within target parameters (between-group comparison);

STUDY FLOW CHART

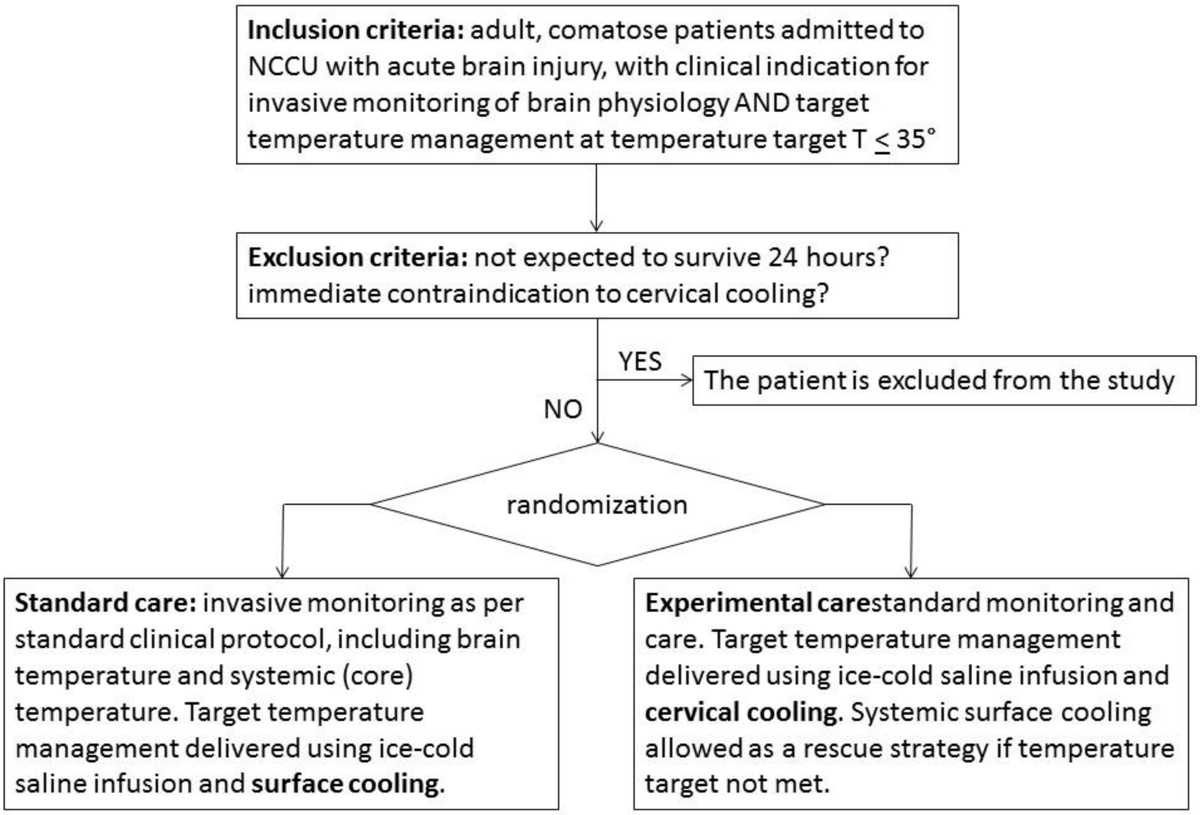

Study Flow Chart. Patient excluded due to immediate contraindications to cervical cooling will be counted. After randomization, patients will be treated and monitored up to 48 hours.

STANDARD CLINICAL PROTOCOL

ICP monitoring as clinically indicated. Primary targets:
ICP $\leq$ 20 mmHg and **CPP 50 to 70 mmHg**
If advanced monitoring is available, fine-tune treatment based on multimodality targets:
PRx < 0.2; **LPR < 25**; **PbtO2 $\geq$ 15 mmHg**

STAGE 1

- **Sedation:** Propofol 2-5 mg/kg/h, Fentanyl 1-4 µg/kg/h; consider Atracurium 0.5 mg/kg/h
- **Ventilation:** $SpO_2 \geq$ 94%; $PaCO_2$ 4.5-5.0 kPa (33-38 mmHg)
- **Circulation:** correct hypovolaemia; vasopressors titrated to CPP > 50 mmHg
- **Temperature:** $\leq$ 37°C (regular paracetamol +/- automated cooling blanket)
- **Nursing:** 15°-30° head up; avoid venous obstruction
- **Nutrition:** early enteral; insulin sliding scale titrated to blood sugar $\leq$ 10 mmol/l
- **DVT prophylaxis:** graduated stockings or intermittent pneumatic compression
- **Antiepileptics:** EEG to esclude seizures +/- institute or escalate antiepileptic therapy

ICP > 20 mmHg? Escalate to **STAGE 2**
consider re-scan +/- SOL evacuation

- **CSF drainage:** open EVD (or consider ventricular catheter insertion)
- **Osmotherapy:** Hypertonic NaCl (up to Na $\simeq$ 160 mmol) or Mannitol (up to 2 ml/kg QDS)
- **Mild hypothermia:** Temp $\simeq$ 35°C (monitor daily lipids, CK and ECG if still on Propofol)

ICP > 20 mmHg? Escalate to **STAGE 3**
consider re-scan +/- SOL evacuation

- **Moderate hypothermia:** Temp 32°C-34°C (change sedation to Midazolam)
- **Ventilation:** $PaCO_2 \simeq$ 4.0 kPa (30mmHg) if SjO2 > 50% and PbtO2 > 15 mmHg

ICP > 20 mmHg? Escalate to **STAGE 4**
consider re-scan +/- **decompression**

- **Burst suppression:** Thiopentone 5 mg/kg + 3-8 mg/Kg/h (titrate to EEG S.R. $\simeq$ 50%)
- **Surgical decompression:** bifrontal or large fronto-temporo-parietal craniectomy

Standard Clinical Protocol. Patients are randomized to standard care or cervical cooling at STAGE 2 of the protocol (yellow box). Physiological data (including ICP, brain oxymetry and L/P metabolic profile) is captured in digital format as per standard practice on our unit.

1. BACKGROUND AND RATIONALE

Targeted temperature management is the standard of care for patients with acute neurological injuries, with level I evidence supporting its use following out of hospital cardiac arrest. The benefits of therapeutic hypothermia in brain injuries associated with trauma, haemorrhage or embolic events remains to be conclusively demonstrated.

It is undisputed that despite the neuroprotective effects of hypothermia, the induction and maintenance of systemic hypothermia is associated with side effects in clinical practice. A recent trial (Eurotherm3235) provides evidence against therapeutic hypothermia as an early measure to lower intracranial pressure [1]. Although the study provides no guidance on the use of therapeutic hypothermia for refractory intracranial hypertension, the findings of the Eurotherm trial raised concerns in regards to whether the risks of therapeutic hypothermia may outweigh its benefits even in patients with refractory intracranial hypertension. It is our opinion that therapeutic hypothermia should remain part of standard clinical protocols for patients with acute brain injuries and intracranial hypertension refractory to “level I” options [2].

Cervical temperature management may provide the benefits of hypothermic or normothermic neuroprotection without the renal, cardiac and infectious side effects of systemic hypothermia. This is a preliminary**, proof-of-concept investigation aiming to demonstrate that cervical cooling can safely induce selective brain cooling.** The study was instigated by the NIHR i4i panel, in consideration of a larger clinical trial of selective brain cooling following severe traumatic brain injury.

Our group previously conducted a preclinical study on large animals (sheep) with a neck cooling prototype, demonstrating that a differential reduction between the brain and body temperature could be induced by cooling the neck of the animal [3]**.** The prototype was also tested on healthy human subjects as part of the development process. In standard conditions (room temperature 23°C, supine resting position) we observed a reduction of tympanic temperature of 1°C, achieved over a period of one hour, followed by the onset of shivering. Testing the strategy in healthy subjects is clearly limited due to the lack of invasive brain temperature monitoring and for limited tolerance to hypothermia in non-sedated patients.

Patients who suffered severe traumatic brain injury are invasively monitored (including brain temperature, core temperature and haemodynamic and brain physiology parameters), they are sedated and they are treated with targeted temperature management as standard of care.

This preliminary study will assess the safety of the strategy of selective brain cooling, including whether the device is able to maintain brain temperature within the target

temperature range set by the treating physician, the type and frequency of side effects or complications associated with its use and its ability to induce and maintain a temperature differential between the brain and the body.

2. OBJECTIVES

2.1 Primary Objective

Safety. Describe number and severity of complications associated with induction and maintenance of hypothermia using selective cervical cooling:

- failure to maintain the brain within target temperature;
- number of instances where external cooling blankets or ice-cold saline had to be used as a rescue strategy to achieve target temperature;
- duration and severity of cerebral hyperthermia;
- any other adverse event;

2.2 Secondary Objectives

Proof of concept. Describe the ability of the device to deliver selective brain cooling, and its effectiveness:

- differential brain-to-core temperature as evidence of selective cooling (between-group comparison);
- brain temperature within target parameters (between-group comparison);

3. STUDY DESIGN

This is a preliminary, proof-of-concept, randomised, interventional study.

The study will enrol at least 10 patients admitted to the Neurosciences and Trauma Critical Care Unit (NCCU, Addenbrooke's) following severe traumatic brain injury requiring invasive brain temperature monitoring and target temperature management as standard of care.

Brain temperature monitoring includes: ICP, brain oxymetry, microdialysis, haemodynamic variables. Physiological parameters and blood tests are also sampled on the ICM+ platform and local electronic medical record systems.

Randomisation of at least 10 patients will be made after the treating clinician has set a target temperature of 35°C. Using predetermined identical envelopes, patients will be assigned to two groups:

Group 1, Standard Care (5 patients): Medical and surgical management as per local treatment protocol (Figure). **Targeted temperature management induced by rapid infusion of ice-cold saline and maintained by automated cooling blankets.**

Group 2, Experimental Care (5 patients): Patients allocated to group 2 will receive the same treatment as those in group 1, with the exception of maintenance of **target temperature by using selective cervical cooling**.

Cervical cooling will be delivered with a purpose built prototype developed by Neuronguard. Brain temperature, core temperature (bladder or oesophageal), ICP and other physiological parameters will be continuously monitored and captured digitally as per standard local practice. Temperature targets are determined by the treating clinician, they will be recorded by a chart at the bedside, updated hourly, with brain temperature targets expressed as absolute values +/- 0.5°C. Monitoring will continue for 48 hours, irrespective of temperature targets (i.e. to include rewarming, if indicated).

3.1 Study Participants

3.2 Inclusion Criteria

Adult, comatose patients admitted to NCCU with acute brain injury as a result of trauma with clinical indication for invasive monitoring of brain physiology (i.e. intracranial pressure, cerebral oximetry and parenchymal brain temperature, cerebral microdialysis) as part of their standard clinical management.

3.3 Exclusion Criteria

Patients with devastating injuries not expected to survive 24 hours will be excluded from the study. Lack of assent from consultee (see informed consent). Contraindications to cervical cooling (for example, an injury limiting access to the neck).

4. STUDY PROCEDURES AND INTERVENTIONS

4.1 Recruitment

Potential participants will be identified at the site of the investigation when they reach "level 2" of the standard clinical protocol (i.e. target temperature 35°C or below). Patients will be identified by the Principal Investigator and data will be captured and stored in our electronic medical record systems.

4.2 Informed Consent

All patients will be comatose and therefore lacking capacity to consent their participation to the study. Whenever possible, a next of kin acting as a "consultee" for the patient will be identified by the research team, and their opinion sought about their perceived participant's wishes and feelings in relation to the participating in the study. A member of the research team will approach the consultee to explain the study and its implications. Subjects are free to withdraw, or be withdrawn by their consultee if appropriate, at any point in the study, and they need not state a reason.
Where it proves impossible to contact consultees either in person, or by telephone, within 48 hours post admission, we will recruit subjects by identifying a nominated consultee, who is not part of the research team, such as the Consultant Neurosurgeon and Critical Care Physician in charge of the patient's care.

4.2 Study Assessments/Interventions

Intervention or procedure	Total number of interventions/procedures received by each participant	Who will conduct the intervention/procedure
Randomisation	1	Principal Investigator / LT
Selective Brain Cooling using the Neuronguard device	1	Principal Investigator / LT
Off-line review of medical data and records for analysis	1	Principal Investigator / LT

4.3 Definition of End of Study

Data collection will be completed after 48 hours of monitoring of patient 10. The end of study is completion of data analysis.

5. SAFETY REPORTING

5.1 Definition of Serious Adverse Events

A serious adverse event is any untoward medical occurrence that:

- Results in death,
- Is life-threatening,
- Requires inpatient hospitalisation or prolongation of existing hospitalisation,
- Results in persistent or significant disability/incapacity, or
- Other important medical events – in this cohort this would be uncontrollable intracranial hypertension

5.2 Reporting Procedures for Serious Adverse Events

A serious adverse event (SAE) occurring to a participant should be reported to the REC that gave a favourable opinion of the study where in the opinion of the Chief Investigator the event was: 'related' – that is, it resulted from administration of any of the research procedures; and 'unexpected' – that is, the type of event is not listed in the protocol as an expected occurrence. Reports of related and unexpected SAEs should be submitted within 15 days of the Chief Investigator becoming aware of the event, using the NRES report of serious adverse event form (see NRES website).

6. STATISTICS

6.1 Number of Participants

This preliminary study will enrol a small number of patients admitted to NCCU, Cambridge, UK. This is a pilot study assessing feasibility and proof-of-concept for a new approach to selective brain cooling, so no formal sample size calculation was indicated. This small scale pilot study was designed based on the indications of the i4i award evaluation Committee that requested the group to assess safety and feasibility of the approach before funding a full scale clinical trial.

6.2 Sampling

We will enrol patients that meet the inclusion criteria consecutively.

6.3 Analysis of Endpoints

Descriptive statistics will be used to report demographics and rate and nature of adverse events.

Non parametric tests will be used for between-group comparison of temperature data e and paired data non parametric statistic tests for the brain/body differential temperature analysis.

Further exploratory data analysis of association between brain physiology, brain temperature, brain-body temperature gradients will be performed in the two groups to generate hypotheses for a larger clinical trial.

7. ETHICAL AND REGULATORY COMPLIANCE

The main ethical challenge is around the issue of consent in comatose patients. Our group has extensive experience on the matter. Whenever possible, a “consultee” is identified and their opinion sought about the potential participant’s wishes and feelings in relation to the project, and whether he or she would have wanted to take part in the study. A member of the research team will approach the consultee to explain the study and its implications. Subjects are free to withdraw, or be withdrawn by their consultee if appropriate, at any point in the study, and they need not state a reason.

In regards to randomization, patients randomized to selective brain cooling using the Neuronguard prototype will be closely monitored and whenever target temperature cannot be achieved (or if there is any deterioration in brain physiology) the treating clinical will resort to systemic cooling, either by rapid infusion of ice cold saline or surface body cooling.

Participants in study group face the risk of lesions on the skin of the neck where cooling is applied. In order to minimize this risk we have created a controlled cooling system that senses the temperature of skin and adjust the heat extraction according to a pre-set level considered safe. The skin of the neck is checked by the research nurse each time the brain and core temperature are measured. In case any sign of lesion is present the treatment is immediately stopped.

7.1 Data Protection and Patient Confidentiality

Data is collected and stored on the Trust safe server. Data exported from the Trust server will be anonymized and stored on encrypted computers. All audio/visual will contain no patient identifiable features. Pictures will be taken to document any adverse reaction connected to this new approach.

The data generated by the study will be analysed on NHS computers of the Cambridge University Hospital by the Principal Investigator and the appointed consultant of bio-statistics (if required). Physiological data will be anonymized and encrypted. Data will be stored in a firewall and password protected, secure NHS drive at the Addenbrooke's Hospital for a minimum of 6 years and possibly indefinitely in accordance with good research practice. Only the Principal Investigator will continue to have access to the data.

Cambridge University Hospital NHS Foundation Trust will keep identifiable information about participants for 5 years after the study has finished.

7.2 Indemnity

NHS indemnity scheme.

8. DISSEMINATION POLICY

The Principal investigator will produce a final study report and draft a paper for submission to a peer reviewed journal, acknowledging the founding bodies. The final study report will be circulated to all participants and clinical and research staff involved with the research. Raw data will be stored on the secure NHS server and owned by the University Department of Anaesthesia.

9. REFERENCES

[1] "Hypothermia for Intracranial Hypertension after Traumatic Brain Injury"
Andrews et Al. NEJM Oct 2015.
https://www.ncbi.nlm.nih.gov/pmc/articles/PMC5138276/

[2] "Correspondence. Hypothermia for Intracranial Hypertension after Traumatic"
Brain Injury.
O'Leary et Al. NEJM April 2016
https://www.nejm.org/doi/pdf/10.1056/NEJMc1600339

[3] "A Novel Cooling Device for Targeted Brain Temperature Control and Therapeutic Hypothermia: Feasibility Study in an Animal Model"
Giuliani at Al. Neurocrit Care 2016
https://www.ncbi.nlm.nih.gov/pmc/articles/PMC5138276/pdf/12028_2016_Article_257.pdf
